# Changes in Core Competencies among Korean University Students Due to Remote Learning during the COVID-19 Pandemic

**DOI:** 10.3390/ijerph18147476

**Published:** 2021-07-13

**Authors:** Sunjoo Jang, Haeyoung Lee

**Affiliations:** Red Cross College of Nursing, Chung-Ang University, Seoul 06974, Korea; icedcoffee@cau.ac.kr

**Keywords:** core competencies, remote learning, online learning, academic performance, problem solving, university students, South Korea, COVID-19

## Abstract

This study aimed to determine the academic performance and learning skills of students who studied through remote teaching methods during the coronavirus disease 2019 (COVID-19) pandemic. It was conducted in February 2021 with 398 university students in South Korea. Data were collected through online surveys. Generalized estimating equations (GEEs) with an autoregressive correlation structure were employed to distinguish differences in core competencies, academic performance, satisfaction, and usefulness of teaching methods before 2019 and after the COVID-19 pandemic. The findings revealed that the overall core competencies of participants were significantly lower in 2020 than before the COVID-19 pandemic. Furthermore, knowledge construction, responsibility practice, and socialization were significantly low during the COVID-19 pandemic, whereas information management and identity value did not show a significant difference. However, problem-solving was higher during the COVID-19 pandemic. Enhancing the core competencies of university students is integral in the new learning environment of the post-COVID-19 era. It is necessary to devise approaches that improve the effectiveness of remote teaching methods and simultaneously augment student satisfaction.

## 1. Introduction

First identified in Wuhan, China, in December 2019, coronavirus disease 2019 (COVID-19) spread globally in a short period and was declared a pandemic by the World Health Organization [1]. Many confirmed cases have occurred in Korea since January 2020 due to mass infection and inflow from other countries, raising the country’s vigilance to the highest level [2]. As of March 2021, there were more than 90,000 confirmed cases in Korea, and approximately 600,000 citizens had been vaccinated, but new cases of infections were still being reported [3].

Students could no longer attend school, and universities had to quickly adopt remote learning since social distancing was implemented to reduce the spread of the virus. Amidst the pandemic, Korean universities uploaded at least 25-min-long video lectures on their servers for students during the first semester of 2020. However, many problems emerged in the process, including complaints about server issues and the quality of video lectures, which led to concerns regarding the learning competencies of university students [4]. Moreover, lack of interaction or difficulty of communication between the instructor and students or among students because of remote classes reduced students’ adjustment to university life and satisfaction [5]. However, the pandemic provided an opportunity to test the capability of learner-centered teaching methods, such as through the integration of the “ubiquitous learning environment” with the university education system [4].

The COVID-19 pandemic enabled the investigation of whether universities were capable of dealing with changes in a crisis. Universities have long emphasized the need to actively cope with changes in the era of the Fourth Industrial Revolution, which will be accelerated based on the “5G network” due to the COVID-19 pandemic [6]. Currently, universities are challenged with the need to prepare for an era of full-fledged distance education [2,4]. In addition to delivering online lectures, teaching methods guiding dynamic learning, such as questions, debates, discussions, explorations, problem-solving, and projects, must be used in remote education to facilitate active interactions and communication among learners, enabling students to control their learning pace and obtain knowledge by studying beforehand [7]. Moreover, a review of studies employing learner-centered pedagogy revealed that learner-centered teaching methods can lead to heutagogy [8].

Various educational achievements attained in classic offline lectures must also be secured through online learning. Therefore, it is necessary to devise mechanisms that evaluate the change in teaching methods according to demand and educational performance. Many universities in Korea utilize only grade point average (GPA) as a key performance indicator of university education and neglect the overall process, such as what students learn and what competencies they develop from university education [9]. Hence, it is imperative to assess core competencies that result in vocational capabilities beyond the assessment of educational performance solely through academic achievement and course satisfaction [10].

Core competencies are defined as abilities necessary to cope with complex changes in life [11]. In particular, core competencies required by university students share the same determinants demanded throughout their lives, but emphasize higher-order capabilities rather than fragmentary knowledge in terms of cognitive competencies, such as the ability to use and restructure knowledge and examine and solve complex problems. Moreover, these capabilities encompass not only cognitive but also affective and social domains, including attitudes, emotions, values, and social and behavioral elements [12]. Therefore, core competencies can evaluate potential knowledge, skills, and attitudes of individual learners and their integration and external manifestation in the form of performance level, serving as a suitable concept for comprehensively evaluating educational performance [12]. However, relatively few studies have determined the relationship between learner characteristics and core competencies of university students to derive implications for university curricula [7,9]. As a paradigm shift in universities is accelerated by the Fourth Industrial Revolution along with the COVID-19 pandemic, it is necessary to gauge the core competencies of university students to understand the current status of university education while preparing for the future.

The tool used in this study to measure the core competencies of university students was applied to evaluate the educational performance and core competencies in several recent studies [13,14,15]. This tool was based on the core competencies described in the OECD’s DeSeCo (Defining and Selecting Key Competencies) project and the K-CESA (Korea Collegiate Essential Skills Assessment) [12]. The core competency described in the DeSeCo project was the ability to cope with important needs in various contexts, in preparation for the rapidly changing modern society [16,17]. As a follow-up study of the DeSeCo project, “OECD Education 2030: The Future of Education and Skills” is in progress. The aim is to find the direction of future social education with the purpose of exploring an educational system that can cultivate competencies required for the society in 2030, and establish an implementation plan for students’ competencies [18]. Therefore, the tool is suitable for measuring the competencies of university students according to the changes in their educational environment accelerated by the COVID-19 pandemic.

Accordingly, this study ascertains the difference between the effect of learning in the past when university students were subjected to traditional teaching methods and the effect of remote learning implemented during the COVID-19 pandemic. We aim to provide basic data for the development of effective teaching methods to prepare for a time when remote learning will be the only way forward. This study aims to determine the academic performance and core competencies of university students studying through a remote teaching method for a year during the COVID-19 pandemic and analyze the difference by comparing it with the academic performance and learning competencies before the pandemic. The specific goals are as follows: (1) distinguish the academic performance, core competencies, and satisfaction and usefulness of teaching methods among university students during the COVID-19 pandemic; (2) evaluate the difference in scores of academic performance, core competencies, satisfaction, and usefulness of teaching methods before and during the COVID-19 pandemic; (3) determine the factors impacting university students’ core competencies during the COVID-19 pandemic.

## 2. Methods

### 2.1. Research Design

We conducted a retrospective study to compare remote learning in 2020 and traditional learning in 2019.

### 2.2. Participants

The effect size (f^2^) of comparing remote learning with traditional learning was small (i.e., 0.1), and a total of 328 participants were included as a result of statistical power analysis (repeated measured analysis of variance [RMANOVA]) utilizing the G*Power 3 program when the power was 0.95 at a significance level of 0.05. The goal was 390 participants, with an expectation that there would be a 15% dropout rate. To compare remote learning experiences with traditional face-to-face teaching methods, the participants had to be (1) university students in South Korea, at least in their sophomore year, (2) studying via traditional face-to-face teaching methods before the COVID-19 pandemic, and (3) receiving remote learning during the COVID-19 pandemic, while (4) understanding the purpose of this study and participating voluntarily. Participants who (1) left school for at least one semester during the years under review or who (2) were students at the Korea National Open University or a cyber university were excluded. The survey was administered in February 2021 through an online survey link, and data were collected from 398 participants.

### 2.3. Measurement Tool

#### 2.3.1. Core Competencies of University Students

The core competency measurement tool was developed based on the tool of Kang et al. [19] and validated to fit the circumstances of Korean university students [12]. This tool can evaluate the individual learner’s latent knowledge, skills, and attitudes, as well as all of their integrated and externally expressed performance-level abilities. The core competency tool for university students [12] constituted 33 items and six categories, such as problem-solving (seven items), knowledge construction (five items), information management (four items), responsibility practice (seven items), identity value (three items), and socialization (seven items). They were rated on a five-point Likert scale ranging from 33 to 165 points, with higher scores indicating higher core competencies. The Cronbach’s ⍺ of the tool was 0.77 [12] when developed. Through a confirmatory factor analysis (CFA), factor loadings were determined to be above 50.

#### 2.3.2. Academic Performance

Academic performance was evaluated using students’ self-reported Grade Point Average (GPA) of the year.

#### 2.3.3. Satisfaction and Usefulness of Teaching Methods

The visual analog scale (VAS) is used to measure psychological phenomena such as satisfaction [20]. Thus, satisfaction and usefulness of teaching methods were measured through this scale (0–10 points).

### 2.4. Data Analysis

The collected data were statistically analyzed utilizing Statistical Package for the Social Sciences (SPSS) version 24.0 (IBM Corp., Armonk, NY, USA). Participants’ general characteristics and learning environment-related characteristics were analyzed using descriptive statistics, namely mean, standard deviation, frequency, and percentage. The Kolmogorov–Smirnov test was used to assess the normality of variables. Differences in core competencies and academic performance according to general characteristics and learning environment-related characteristics were analyzed using *t*-tests and ANOVA. The relationships between age, core competencies, and academic performance were analyzed using Pearson’s correlation coefficient. The clustered correlated data obtained were measured twice and the generalized estimating equations (GEEs), with an autoregressive correlation structure, were used to distinguish the differences in core competencies, academic performance, satisfaction, and usefulness of teaching methods before and during the COVID-19 pandemic. GEEs are actively used in nursing as they do not assume independence and homogeneity of variance and can control confounders [21]. Lastly, Cronbach’s α was calculated to test tool reliability.

## 3. Results

### 3.1. The Reliability of Core Competencies of University Students

The Cronbach’s ⍺ was 0.93 in this study, and the coefficients of the subcategories ranged from 0.70–0.83.

### 3.2. General Characteristics of Participants and Characteristics Related to the Learning Environment

Table 1 shows the 398 participants’ general and learning environment-related characteristics. The mean age was 23.14 years; 50.8% were female, 50.3% were seniors, and 42.5% were majoring in humanities and social sciences. In total, 84.9% were evaluated using relative grading during the COVID-19 pandemic. Notably, several participants (61.6%) used laptops for remote learning.

### 3.3. Differences in Core Competencies and Academic Performance during the Coronavirus Disease 2019 (COVID-19) Pandemic due to Participants’ Characteristics

Table 2 shows the differences in core competencies and academic performance during the COVID-19 pandemic according to participants’ characteristics. No significant difference in core competencies due to general and learning environment characteristics was evident during the COVID-19 pandemic, but GPA was much lower in male students than in female students (*t* = 2.25, *p* = 0.025).

### 3.4. Correlation between Participant Characteristics, Core Competencies, and Academic Performance during the COVID-19 Pandemic

Table 3 shows the results of the Pearson correlation analysis for participant characteristics, core competencies, and academic performance. Age signified a weak negative correlation with socialization (*r* = −0.102, *p* = 0.042), and overall core competencies highlighted a positive correlation with six subcategories (*r* = 0.699–0.872, *p* < 0.001) and GPA (*r* = 0.281, *p* < 0.001). Specifically, GPA was positively correlated with information management (*r* = 0.198, *p* < 0.001), knowledge construction (*r* = 0.234, *p* < 0.001), problem-solving (*r* = 0.162, *p* < 0.001), identity value (*r* = 0.244, *p* < 0.001), responsibility practice (*r* = 0.355, *p* < 0.001), and socialization (*r* = 0.168, *p* = 0.001).

### 3.5. Comparison of Differences in Core Competencies, Academic Performance, Satisfaction, and Usefulness of Teaching Methods Before and Amid the COVID-19 Pandemic

The results of comparing the differences in core competencies, academic performance, satisfaction, and usefulness of teaching methods before and amid the COVID-19 pandemic using GEE are summarized in Table 4. The overall core competencies of the participants were significantly lower in 2020 than before the COVID-19 pandemic (*B* = −1.13, *SE* = 0.47). Among the subcategories, knowledge construction (*B* = −0.38, *SE* = 0.13), responsibility practice (*B* = −0.57, *SE* = 0.13), and socialization (*B* = −0.38, *SE* = 0.12) were also significantly lower in 2020 than in 2019. However, problem-solving (*B* = 0.35, *SE* = 0.16) was higher during the COVID-19 pandemic, and there was no significant difference in information management and identity value. GPA was higher during the COVID-19 pandemic (*B* = 0.20, *SE* = 0.02), but satisfaction with teaching methods (*B* = −0.83, *SE* = 0.09) and usefulness (*B* = −1.01, *SE* = 0.11) were lower during 2020.

## 4. Discussion

This study identified the academic performance level and core competencies of university students studying via remote teaching methods during the COVID-19 pandemic and analyzed the differences in their academic performance and core competencies before the pandemic. The overall core competencies of participants were significantly lower in 2020 than in 2019. The subcategories of core competencies, namely knowledge construction, responsibility practice, and socialization, were significantly lower during rather than before the COVID-19 pandemic. Nonetheless, problem-solving was higher during the COVID-19 pandemic, and information management and identity values did not denote a significant difference. A previous study assessing the core competencies of university students subjected to conventional teaching methods outlined that problem solving was relatively lower than other competencies [12], but this study revealed that problem-solving was higher during the pandemic. Problem-solving refers to inspecting and reflecting on critical aspects of everyday problems and finding strategies to solve them using optimal methods based on comprehensive thinking of the given situation [22]. The aforementioned outcome is positive, showing the possibility that problem-solving, which is rated more highly in remote learning, can address the lack of development concerning higher-order thinking among learners, which has been deemed a limitation under conventional learning methods [23]. The utilization of the Internet influences cognitive processes, making learners accept incoming information in a multifaceted manner; however, unlike online learning, the results of the evaluation may appear to be different when the learners are forced to participate in online learning due to an external factor [12]. Hence, closely analyzing why problem-solving during the COVID-19 pandemic was rated more highly than under conventional teaching methods is necessary.

Interactions between the instructor and learners in an ideal class environment increase academic achievement and satisfaction in learning [24], and learners who interact more actively have greater problem-solving skills [25]. Further, academic stress is a factor that impacts problem-solving, which can be managed by increasing interactions and intimacy between the instructor and learners [26]. Considering that the satisfaction and usefulness of remote learning during the COVID-19 pandemic indicated relatively poor scores, it is necessary to promote interactions between the instructor and learners to increase student satisfaction and learning motivation. Various teaching methods must be applied aside from lectures so that students can solve problems themselves by choosing and constructing the information they need [12].

This study revealed lower satisfaction and usefulness of teaching methods during the COVID-19 pandemic. Evaluation of remote education in universities due to the COVID-19 pandemic has been reported in many studies worldwide [4,27,28,29,30]. A study in Canada observed a decrease in university students’ achievement goals, engagement, and perceptions of success, and an increase in perceptions of cheating [28]. In a study in India, a majority of students responded that they liked receiving remote education during the lockdown, but they reacted negatively toward maintaining the same education method after the lockdown was lifted, responding that the teaching method before the COVID-19 pandemic was better in many ways (i.e., topic understanding and net connectivity) [31]. This research presented lower scores for the satisfaction and usefulness of teaching methods during the COVID-19 pandemic because instructors and learners were not used to the new platform as the system for remote learning was not perfectly prepared, or because there were insufficient instructor–learner interactions, which decreased learning motivation [32].

As previously mentioned, learning motivation is an essential variable in predicting the success of online learning. Higher learning motivation results in more active participation in online learning, raising the chances for success [32]. A previous study implementing remote classes for a majority of courses related to nursing science due to the COVID-19 pandemic also proved that learning motivation has the greatest impact on students’ problem-solving skills and academic satisfaction [33]. With regard to remote education, self-regulated learning is pivotal as learners take responsibility for their learning process and participate actively. Students with greater self-regulated learning skills obtained more satisfactory results and continued their studies [34]. Still, students did not opt for online learning but were forced to accept it during the COVID-19 pandemic, which may have hindered their motivation for learning [32]. Mastery goals have significant relevance to positive learning processes and outcomes, including positive emotions, interest, persistence, effective self-regulation, and learning strategies [35]. Considering that motivational and self-regulated learning is a key factor affecting students’ academic achievement [36], it is important to not only prepare a remote learning system but also support students to enhance learning motivation and engagements in self-regulated learning.

During the lockdown, there was no significant difference in core competencies due to the general characteristics of the participants and learning environment characteristics. Past studies regarding the relationship between university students’ core competencies and other variables determined that core competencies varied depending on gender [10], and they may also increase with a higher academic level [37]. Few studies noticed that there was a difference in core competencies [10], while others detected no significant difference [38]. Core competencies are not just cultivated by the stages of lifelong development but are influenced by the curricula of each university or various life experiences of individual learners. Therefore, it can be predicted that there would be diverse levels of core competencies among groups with all kinds of individuals [12]. These researchers also attained slightly different outcomes in terms of differences in core competencies due to the general characteristics of participants, and further research is necessitated. In particular, this study did not show a significant difference in core competencies due to learning environment characteristics since many students already had a learning space and device to listen to online lectures. Altogether, 92.7% of the participants had access to personal space for online learning, and 100% had the environment and device to use the Internet. Considering that Korea has the highest smartphone ownership and Internet usage in the world [39], and university students who participated in the study were digital natives born in the late 1990s to the early 2000s, also classified as Generation Z [40], learning environment characteristics may not have affected core competencies, even with the sudden change in the learning environment due to the COVID-19 pandemic.

This study had several limitations. First, as a cross-sectional descriptive study, it is impossible to explain the causal relationship between remote teaching methods adopted during the COVID-19 pandemic and the academic performance and core competencies of university students. In addition, we cannot rule out the maturation effect because a difference-in-difference estimation has not been applied in this study. Thus, a longitudinal study must be conducted to validate how the application of remote teaching methods affects students’ academic performance and core competencies. Second, academic performance level and core competencies gauged in this study are based on students’ responses in the form of a self-report questionnaire through a retrospective study, which leaves the possibility of recall bias. Therefore, future research must rely on observations or multidimensional assessments for the objective evaluation of students’ core competencies. Third, although time series comparisons were made using GEE, the impact of differences in propensity based on the subjects’ general characteristics on the results has not been considered. In addition, for the competency variables, interactions and collinearity amongst these variables were not examined. Fourth, this research was a quantitative study that depended on a survey to evaluate satisfaction and usefulness; hence, a qualitative study is required for a more comprehensive analysis of student demands. Finally, this study intended to include various participants through an online survey, but the samples do not represent all university students; thus, the results cannot be generalized. Accordingly, a replication study is recommended to generalize the results. However, despite these limitations, this study identified and examined the core competencies of university students via six factors amid the changing process of university education due to COVID-19. Therefore, it provides basic data for the development of teaching and evaluation methods to enhance the core competencies of students during post-COVID-19 university education.

## 5. Conclusions

The health, social, and economic crisis caused by the COVID-19 pandemic further highlighted the essential role that information and communication technologies (ICT) currently play. Moreover, the social isolation caused by the pandemic forced educational institutions (EI) to reinvent themselves in a short period of time. If on the one hand, the pandemic forced EI to adapt quickly to the new conditions for teaching and learning, on the other hand, it has also provided an opportunity to test the capacity of student-centered teaching methods. In this study, the core competencies of university students who learned through remote teaching methods during the COVID-19 pandemic were identified and compared with the effects of traditional teaching methods prior to the pandemic. The results demonstrated that the overall core competency of the participants was significantly lower in 2020, as compared to 2019. However, problem-solving capabilities were higher during the COVID-19 pandemic.

There are several limitations in generalizing the higher evaluation of problem-solving in this study, to the overall effects of remote learning. However, the glimpse of a positive possibility that students’ problem-solving ability was higher in the chaotic era bears witness to the welcome given to a new era, which is a meaningful result of our study. Remote learning has become inevitable due to the COVID-19 pandemic. Even if the world overcomes COVID-19 with vaccinations, remote learning will continue to be applied in various ways. During this transition wherein university education is facing a remarkable change, it is important to identify how remote learning impacts the competency development of students and steers the education system toward a more progressive and developmental direction. The satisfaction and usefulness of the teaching methods were also rated low during the COVID-19 pandemic, since remote learning methods are not yet stabilized as a universal education system. Therefore, it is imperative to sufficiently decipher the needs of students and analyze the pros and cons of current university education. This would help to create an education system that can satisfy both students and professors while enhancing the students’ core competencies.

## Figures and Tables

**Table 1:** General characteristics and characteristics related to the learning environment.

Variables	Categories	N (%)	M (SD)
Gender	Male	196 (49.2)	
	Female	202 (50.8)	
Age (years)			23.14 (2.21)
Year	2	78 (19.6)	
	3	120 (30.2)	
	4	200 (50.3)	
Major	Humanities and social sciences	169 (42.5)	
	STEM	142 (35.7)	
	Health and medical studies	42 (10.6)	
	Art and physical education	28 (7.0)	
	Other	17 (4.3)	
Socioeconomic status	Low	31 (7.8)	
	Medium-low	99 (24.9)	
	Medium	166 (41.7)	
	Medium-high	93 (23.4)	
	High	9 (2.3)	
Personal space to study	Yes	369 (92.7)	
	No	29 (7.3)	
Evaluation method for 2020	Absolute grading	60 (15.1)	
	Relative grading	338 (84.9)	
Device mainly used to attend online video classes	PC	112 (28.1)	
	Tablet PC	25 (6.3)	
	Laptop	245 (61.6)	
	Cell phone	16 (4.0)	
Ownership of any device	Personally owned	392 (98.5)	
	Shared	6 (1.5)	
Internet environment	Public wi-fi	50 (12.6)	
	Private wi-fi	348 (87.4)	
Access to internet connection VAS (0–10)			7.69 (1.76)

M: mean, SD: standard deviation, STEM: science, technology, engineering, and mathematics, PC: personal computer, VAS: visual analogue scale.

**Table 2:** The difference in core competencies and academic performance according to participant characteristics.

Variables	Categories	Core Competencies (2020)		GPA (out of 4.50) (2020)	
		M (SD)	t/F (*p*)	M (SD)	t/F (*p*)
Gender	Male	122.37 (16.90)	1.88 (0.060)	3.85 (0.49)	2.25 (0.025) *
	Female	125.56 (16.94)		3.96 (0.43)	
Year	2	125.97 (17.39)	2.16 (0.117)	3.82 (0.56)	2.45 (0.088)
	3	125.62 (15.40)		3.97 (0.40)	
	4	122.24 (17.60)		3.90 (0.46)	
Major	Humanities and social science	123.54 (17.49)	2.02 (0.092)	3.94 (0.45)	1.88 (0.113)
	STEM	122.04 (15.48)		3.84 (0.50)	
	Health and medical studies	129.79 (19.20)		3.98 (0.39)	
	Art and physical education	125.61 (14.92)		3.88 (0.45)	
	Other	127.76 (19.09)		4.08 (0.37)	
Socioeconomic status	Low	127.77 (20.67)	2.06 (0.086)	3.87 (0.54)	0.55 (0.699)
	Medium-low	120.72 (16.94)		3.85 (0.49)	
	Medium	124.07 (15.92)		3.93 (0.41)	
	Medium-high	127.61 (16.26)		3.93 (0.48)	
	High	125.33 (24.97)		3.96 (0.51)	
Personal space to study	Yes	124.26 (16.47)	0.88 (0.388)	3.91 (0.47)	0.02 (0.986)
	No	120.52 (22.57)		3.91 (0.42)	
Evaluation method for 2020	Absolute grading	123.72 (15.58)	0.14 (0.893)	3.88 (0.49)	0.50 (0.617)
	Relative grading	124.04 (17.23)		3.91 (0.46)	
Device mainly used to attend remote classes	PC	123.51 (16.61)	0.22 (0.885)	3.89 (0.52)	1.33 (0.263)
	Tablet PC	124.24 (18.17)		3.74 (0.54)	
	Laptop	124.37 (16.75)		3.93 (0.42)	
	Cell phone	121.19 (22.03)		3.91 (0.51)	
Ownership of device	Personally owned	124.12 (16.88)	1.26 (0.209)	3.91 (0.46)	0.03 (0.974)
	Shared	115.33 (22.74)		3,91 (0.52)	
Internet environment	Public wi-fi	125.14 (16.03)	0.51 (0.609)	3.99 (0.40)	1.29 (0.197)
	Private wi-fi	123.82 (17.13)		3.90 (0.47)	
Access to internet Connection	Difficult	121.24 (15.57)	1.17 (0.243)	3.86 (0.42)	0.76 (0.447)
	Easy	124.35 (17.14)		3.91 (0.47)	

GPA: grade point average, M: mean, SD: standard deviation, STEM: science, technology, engineering, and mathematics, PC: personal computer, * *p* < 0.05.

**Table 3:** Correlation among participant characteristics, core competencies, and academic performance.

	Age	Core Competency	Information Management	Knowledge Construction	Problem-Solving	Identity Value	Responsibility Practice	Socialization	GPA
	r(*p*)	r(*p*)	r(*p*)	r(*p*)	r(*p*)	r(*p*)	r(*p*)		r(*p*)
Age	1								
Core competency	−0.047 (0.348)	1							
Information management	−0.046 (0.360)	0.710 *** (<0.001)	1						
Knowledge construction	0.003 (0.946)	0.770 *** (<0.001)	0.548 *** (<0.001)	1					
Problem-solving	0.055 (0.270)	0.775 *** (<0.001)	0.408 *** (<0.001)	0.595 *** (<0.001)	1				
Identity value	−0.110 * (0.028)	0.705 *** (<0.001)	0.515 *** (<0.001)	0.388 *** (<0.001)	0.385 *** (<0.001)	1			
Responsibility practice	−0.056 (0.261)	0.872 *** (<0.001)	0.507 *** (<0.001)	0.575 *** (<0.001)	0.600 *** (<0.001)	0.612 *** (<0.001)	1		
Socialization	−0.102 * (0.042)	0.699 *** (<0.001)	0.395 *** (<0.001)	0.381 *** (<0.001)	0.439 *** (<0.001)	0.592 *** (<0.001)	0.619 *** (<0.001)	1	
GPA	−0.042 (0.400)	0.281 *** (<0.001)	0.198 *** (<0.001)	0.234 *** (<0.001)	0.162 *** (<0.001)	0.244 *** (<0.001)	0.355 *** (<0.001)	0.168 ** (0.001)	1

GPA: grade point average, * *p* < 0.05, ** *p* < 0.01, *** *p* < 0.001.

**Table 4:** Comparison of differences in core competencies, academic performance, satisfaction, and usefulness of teaching methods between before and during the COVID-19 pandemic.

	95% Wald CI					
	*B*	*SE*	Lower	Upper	Wald χ^2^	*p*
Core Competency						
COVID-19 pandemic	−1.13	0.47	−2.05	−0.21	5.75	0.016 *
a. Information management						
COVID-19 pandemic	−0.03	0.12	−0.27	0.21	0.07	0.789
b. Knowledge construction						
COVID-19 pandemic	−0.38	0.13	−0.64	−0.12	8.10	0.004 **
c. Problem solving						
COVID-19 pandemic	0.35	0.16	0.05	0.64	5.23	0.022 *
d. Identity Value						
COVID-19 pandemic	−0.11	0.06	−0.24	0.01	3.05	0.081
e. Responsibility practice						
COVID-19 pandemic	−0.57	0.13	−0.83	−0.31	18.81	<0.001 ***
f. Socialization						
COVID-19 pandemic	−0.38	0.12	−0.62	−0.13	9.18	0.002 **
Academic performance (GPA)						
COVID-19 pandemic	0.20	0.02	0.16	0.23	103.19	<0.001 ***
Satisfaction with teaching methods						
COVID-19 pandemic	−0.83	0.09	−1.02	−0.64	76.78	<0.001 ***
Usefulness of teaching methods						
COVID-19 pandemic	−1.01	0.11	−1.22	−0.80	86.61	<0.001 ***

Note. Covariates: sex, age, year, socioeconomic status, major, personal space to study, absolute grading/relative grading system, device mainly used, ownership of device, internet environment, access to internet connection; SE, standard error; CI, confidence interval; *p*-value: GEE model adjusted for covariates * *p* < 0.05, ** *p* < 0.01, *** *p* < 0.001.

## Data Availability

The data presented in this study are available on request from the corresponding author and with permission from the Institutional Review Board of C University.
